# Longitudinal changes in functional connectivity and pain-induced brain activations in patients with migraine: a functional MRI study pre- and post- treatment with Erenumab

**DOI:** 10.1186/s10194-022-01526-5

**Published:** 2022-12-14

**Authors:** Todd J. Schwedt, Simona Nikolova, Gina Dumkrieger, Jing Li, Teresa Wu, Catherine D. Chong

**Affiliations:** 1 Department of Neurology, Mayo Clinic, Phoenix, AZ USA; 2 School of Industrial and Systems Engineering, Georgia Tech, Atlanta, GA USA; 3 School of Computing, Informatics, Decision Systems Engineering, Arizona State University, Tempe, AZ USA

**Keywords:** Calcitonin gene-related peptide, Monoclonal antibody, Magnetic resonance imaging, Headache, Functional connectivity, Graph theory, Brain networks

## Abstract

**Abstract:**

**Background:**

Migraine involves central and peripheral nervous system mechanisms. Erenumab, an anti-calcitonin gene-related peptide (CGRP) receptor monoclonal antibody with little central nervous system penetrance, is effective for migraine prevention. The objective of this study was to determine if response to erenumab is associated with alterations in brain functional connectivity and pain-induced brain activations.

**Methods:**

Adults with 6–25 migraine days per month during a 4-week headache diary run-in phase underwent pre-treatment brain functional MRI (fMRI) that included resting-state functional connectivity and BOLD measurements in response to moderately painful heat stimulation to the forearm. This was followed by two treatments with 140 mg erenumab, at baseline and 4 weeks later. Post-treatment fMRI was performed 2 weeks and 8 weeks following the first erenumab treatment. A longitudinal Sandwich estimator analysis was used to identify pre- to post-treatment changes in resting-state functional connectivity and brain activations in response to thermal pain. fMRI findings were compared between erenumab treatment-responders vs. erenumab non-responders.

**Results:**

Pre- and post-treatment longitudinal imaging data were available from 32 participants. Average age was 40.3 (+/− 13) years and 29 were female. Pre-treatment average migraine day frequency was 13.8 (+/− 4.7) / 28 days and average headache day frequency was 15.8 (+/− 4.4) / 28 days. Eighteen of 32 (56%) were erenumab responders. Compared to erenumab non-responders, erenumab responders had post-treatment differences in 1) network functional connectivity amongst pain-processing regions, including higher global efficiency, clustering coefficient, node degree, regional efficiency, and modularity, 2) region-to-region functional connectivity between several regions including temporal pole, supramarginal gyrus, and hypothalamus, and 3) pain-induced activations in the middle cingulate, posterior cingulate, and periaqueductal gray matter.

**Conclusions:**

Reductions in migraine day frequency accompanying erenumab treatment are associated with changes in resting state functional connectivity and central processing of extracranial painful stimuli that differ from erenumab non-responders.

**Trial registration:**

clinicaltrials.gov (NCT03773562).

## Introduction

Prior publications have demonstrated abnormal brain structure, functional connectivity, and stimulus-induced brain responses amongst those with migraine [1–6]. Other studies have demonstrated cycling activity and functional connectivity of brain regions such as the hypothalamus, pons, and trigeminal nucleus, that correlate with the occurrence of migraine attacks [3, 4, 7]. Only a few studies have investigated changes in resting-state functional connectivity or pain-induced brain activations associated with response to migraine preventive treatment [8–12]. These publications demonstrate that migraine preventive treatments, even those that are unlikely to directly access the brain, may be associated with changes in brain functional connectivity and pain-induced activations.

Erenumab (erenumab-aooe in the U.S.), a monoclonal antibody (mAb) that targets the calcitonin gene-related peptide (CGRP) receptor, is effective for the prevention of episodic and chronic migraine [13–15]. The anti-CGRP pathway monoclonal antibodies are very large molecules that have little penetrance through the blood-brain barrier and therefore are not thought to exert their effects *directly* in the central nervous system. None-the-less, they are effective migraine preventive medications, possibly through their action at locations outside of the blood-brain barrier such as the trigeminal ganglia, trigeminal nerves, or dura mater [16–18]. A previously published functional magnetic resonance imaging (fMRI) study demonstrated that erenumab treatment is associated with changes in central processing of trigeminal pain and brain resting state functional connectivity at 2 weeks after starting treatment [10]. Another study investigated the impact of treatment with galcanezumab, another anti-CGRP pathway mAb, on brain activation in response to trigeminal nociception measured 2–3 weeks after starting galcanezumab [12]. The objective of our study was to determine if participants with migraine who respond to erenumab, an anti-CGRP pathway mAb targeting the receptor, have post-treatment changes in functional connectivity and pain-induced brain activations that differ from erenumab non-responders. To address this objective, we interrogated 1) resting-state functional connectivity amongst brain regions involved with pain processing, including global and regional graph theory network measures and region-to-region connectivity, and 2) brain responses to extracranial painful thermal stimuli. Furthermore, our study investigated the timing of these fMRI changes, with measurements available from 2 weeks and 8 weeks following the first erenumab treatment.

## Methods

### Informed consent and study registration

This study was approved by the Mayo Clinic Institutional Review Board. Each research participant reviewed and signed a consent form after participating in the informed consent process. The study was registered at clinicaltrials.gov (NCT03773562).

### Eligibility criteria

Adults between the ages of 18–65 years who had episodic migraine or chronic migraine and self-reported a history of 6–25 migraine days per month during the prior 3 months were eligible for participation. Migraine diagnoses were made according to the International Classification of Headache Disorders 3rd edition criteria [19]. Individuals were excluded if they were older than 50 years of age at migraine onset, had migraine onset within the prior 12 months, had cluster headache or hemiplegic migraine, continuous headache (i.e. no headache free periods during the 1 month prior to screening), used opioids or butalbital on 6 or more days per month, had no therapeutic response to adequate trials of migraine preventive medications from four or more different medication classes, were currently taking more than two migraine preventive medications, had received botulinum toxin treatment within the prior 4 months or nerve blocks used for treatment of headache within 2 months, had a history of myocardial infarction, stroke, transient ischemic attack, unstable angina, coronary artery bypass surgery, or other revascularization procedures within the prior 12 months, had contraindications to MRI, were pregnant or lactating, were not willing to use a reliable form of contraception, or had received a CGRP pathway mAb within the prior 4 months. After completing the four-week run-in diary phase, those who had between 6 and 25 migraine days and were at least 80% compliant with providing headache diary data were eligible to continue in the study.

### Research procedures

For those participants who qualified for continued study participation after the run-in phase, there were a total of six research visits during a 16-week period. (Table 1) Questionnaires were completed at each visit and a headache diary was maintained for the entire 16 weeks. Brain MRIs and quantitative sensory testing (QST) were performed three times. Each participant received two treatments with 140 mg of erenumab. Adverse events (AEs) were collected through 12 weeks following the first erenumab treatment.

**Table 1:**
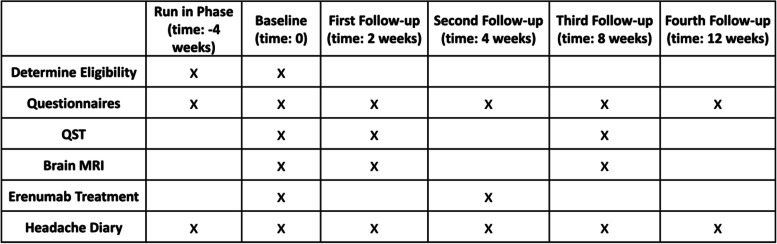
Research Visits and Activities. Only those participants who continue to meet eligibility criteria after the run-in phase participated in the subsequent research visits. QST = quantitative sensory testing; MRI = magnetic resonance imaging

Questionnaires and Structured Interviews: Structured interviews and questionnaires were used to collect data on participant demographics, migraine history and characteristics, medications, medical history, migraine-related disability (Migraine Disability Assessment (MIDAS)), depression (Beck Depression Inventory (BDI)), and cutaneous allodynia (Allodynia Symptom Checklist – 12 (ASC-12)) [20–22]. Adverse events were collected at each study visit.

Headache Diary: The headache diary was an e-diary that was developed within REDCap [23]. Each day, participants were prompted to provide information about the presence of headache and associated symptoms. The headache diary was used to determine the number of headache days and migraine days experienced by each participant. A migraine day was defined as any calendar day during which a person experienced a qualified migraine headache (onset, continuation, or recurrence of the migraine headache). A qualified migraine headache was defined as a migraine with or without aura, lasting for at least 30 minutes, and meeting at least one of the following criteria (a and/or b): a) at least 2 of the following pain features: unilateral, throbbing, moderate to severe, exacerbated with exercise/physical activity; b) at least 1 of the following associated symptoms: nausea and/or vomiting, photophobia and phonophobia. If the participant took a migraine-specific as-needed medication (e.g., triptan or ergotamine) on a calendar day, then it was counted as a migraine day regardless of the duration and pain features/associated symptoms.

Quantitative Sensory Testing (QST): A Medoc Pathway platform with a 30 mm × 30 mm thermode was used for QST to determine the temperature required to cause moderately intense heat pain. Moderately intense heat pain was defined by a participant rating their pain intensity between 4 and 7, ratings consistent with moderate intensity pain, using an 11-point scale in which zero indicates no pain and 10 indicates the most severe pain. Initially, heat pain threshold was determined for each participant using the method of limits. The thermode was placed on the left forearm and fastened with a Velcro strap. The thermode increased in temperature from 32 degrees Celsius at a rate of one degree Celsius per second until the participant pushed a button indicating that the stimulus changed from a heat sensation to a painful sensation. The average of three trials was considered the heat pain threshold. Participants were then stimulated for 7.5 seconds with a temperature equal to their heat pain threshold plus 1 degree Celsius. Individuals then rated the pain intensity. If pain intensity was rated below 4, the temperature was increased in 0.5 degree Celsius increments until rated between 4 and 7. Conversely, if the pain intensity was rated greater than 7, the stimulation temperature was decreased in 0.5 degree Celsius increments until the participant rated the pain between 4 and 7/10 in intensity. QST methods were consistent with those previously reported [24].

Brain MRI: Brain MRIs were completed on a Siemens Skyra 3 T scanner. Sequences included a high-resolution 3D T1-weighted MPRAGE sequence (TE 3.03 ms, TR 2400 ms, voxel size 1x1x1.25 mm, field of view 256x256x160 mm, flip angle 8 degrees), two five-minute runs of resting-state blood-oxygen-level-dependent (BOLD) collection with the participant relaxing with eyes closed (TE 27 ms, TR 2500 ms, voxel size 4x4x4 mm, field of view 256x256x152 mm, flip angle 90 degrees), and three runs of event-related BOLD collection (TE 27 ms, TR 2500 ms, voxel size 4x4x4 mm, field of view 256x256x152 mm, flip angle 90 degrees) during which participants received painful thermal stimuli. During the event-related paradigm, the thermode was attached to the left forearm with a Velcro strap. An auditory cue was followed by moderately intense heat pain, using the temperature determined to cause moderately intense pain via testing of the participant just before the MRI. Heat pain stimuli were delivered nine times for each patient, with each stimulus lasting for 7.5 seconds and the interstimulus interval varying from 44 to 46 seconds. Auditory cue followed by no thermal stimulus (i.e., no change in the thermode temperature) was randomly inserted into the event-related paradigm to account for brain activations associated with pain anticipation or alerting responses. The event-related paradigm methods were consistent with those previously reported [24].

Erenumab Treatment: Participants received treatment with 140 mg of erenumab via subcutaneous injection into the upper arm. Injections were completed in the office during two research visits separated by 4 weeks.

### Pain-induced activation MRI data processing

All preprocessing and General Linear Model (GLM) estimation of whole-brain activation patterns were performed using SPM12 software (Wellcome Department of Cognitive Neurology, Institute of Neurology, London, UK) interfaced with MATLAB version 11.0 (Mathworks, Natick, MA, USA). Functional images were realigned to the mean volume in the series, motion-corrected, realigned to each individual’s structural images, and smoothed using an 8 mm Full Width Half Maximum (FWHM) Gaussian kernel. Data were checked for excessive motion and all included scans showed < 3 mm movement in any direction. First-level single subject analysis was performed within SPM12 using a GLM approach with regression of 6 motion parameters. The immediate BOLD response to pain stimuli used an event-related design with the measured duration of hemodynamic response equal to 12.5 s (i.e. five TRs), starting with the MRI frame during which the painful heat stimulus began and ending 12.5 s later. The delayed response to pain was also investigated, with measurements starting 12.5 s after onset of painful heat stimulus and ending 12.5 s later [25]. Brain regions activated in response to painful stimuli were identified by generating contrast maps representing activations associated with auditory cue followed by painful stimulation vs. activations associated with auditory cue followed by no stimulation. Each individual’s contrast map was normalized to standard stereotaxic space using the Montreal Neurological Institute template (MNI 152).

Statistical analysis was performed using the Sandwich estimator (SWE) for neuroimaging longitudinal data toolbox version 1.2.8 SWE (SWE, Guillaume 2014 & 2015) interfaced with MATLAB [26]. All contrast maps were added to the SWE longitudinal model with default settings for small sample adjustments and estimated degrees of freedom. Responder vs non-responder group-visit effects were examined using F-statistic to include positive and negative relationships. For the pain vs no stimulation contrast, a false discovery rate with *p* < 0.05 with a cluster forming threshold of 10 voxels was set for the main effect and uncorrected *p* < 0.005 with a cluster forming threshold of 50 voxels for the group visit effect [27]. The delayed response used an uncorrected *p* < 0.001 with a cluster forming threshold of 10 voxels for the main effect and uncorrected p < 0.005 with a cluster forming threshold of 25 voxels for the group visit effect.

### Resting functional connectivity MRI data processing

Resting-state functional MRI data were pre-processed using standard procedures in SPM12 including the following steps: slice-time correction, motion correction, re-alignment to first image of the set, re-alignment to an average MNI template, and smoothing with a 6 mm FWHM Gaussian kernel. Further post-processing in AFNI 3dTproject included band-pass filtering (0.01–0.1 HZ) after removal of nuisance signals from framewise displacement, cerebrospinal fluid signal, and linear drift [28].

A region of interest approach (ROI) was used to interrogate functional connectivity. Thirty-one bilateral ROIs and one midline ROI were selected based on previous findings demonstrating that these regions participate in pain processing, migraine, and/or multisensory integration [3, 7, 10, 29–36]. [Table 2] Each ROI was an 8 mm diameter sphere created using MarsBaR toolbox in SPM12. The Pearson correlation coefficient was calculated between the signals of the ROI pairs where motion did not exceed 2 mm. The first five frames were excluded to allow the signal to reach steady state. Following the correlation analysis, Fisher r-z transforms were calculated for each ROI-to-ROI pair and third visit mean functional connectivity was compared between erenumab-responders and non-responders using two tailed t-tests (uncorrected *p*-value less than 0.005 considered significant). An undirected weighted adjacency matrix was then created for each participant with ROI-ROI z-scores as the edge weights. The diagonal was set to zero to exclude self-connections. Nodes with z-scores less than 0.1 were set to zero to construct undirectional weighted functional connectomes. Global graph theory measurements were performed for global efficiency [37]. Local measurements were performed for efficiency, clustering coefficient, degrees centrality (betweenness and degree), and modularity [38–40]. Two sample t-tests (2 tailed) were performed comparing erenumab-responders to non-responders at the 3rd visit with significance defined as an uncorrected *p*-value less than 0.05.

**Table 2:** Regions of Interest. The 63 a priori-selected ROIs and their MNI coordinates are shown. For thirty-one of the ROIs, a right-hemisphere and a left-hemisphere ROI was included. Only a midline ROI for periaqueductal gray was included. Each ROI was an 8 mm diameter sphere

Region Name	MNI Coordinates (X, Y, Z)		
Anterior Insula	+/− 38	19	−3
Anterior Cingulate	+/− 6	28	24
Middle Cingulate	+/− 10	−7	46
Posterior Cingulate	+/− 8	− 48	39
Posterior Insula	+/− 40	− 14	1
Thalamus	+/− 8	− 21	7
Primary Somatosensory	+/− 46	−24	47
Dorsolateral Prefrontal	+/− 40	39	24
Inferior Lateral Parietal	+/− 57	−48	30
Ventromedial Prefrontal	+/− 6	36	−14
Secondary Somatosensory	+/− 52	−28	21
Somatomotor	+/− 6	1	68
Temporal Pole	+/− 41	10	−32
Amygdala	+/− 22	−1	−22
Middle Temporal	+/− 60	−26	−5
Caudate	+/− 14	13	11
Middle Occipital	+/− 34	− 72	6
Cuneus	+/− 13	− 93	9
Hypothalamus	+/− 6	−6	−12
Lingual Gyrus	+/− 19	− 64	−11
Spinal Trigeminal Nucleus	+/− 6	− 39	− 45
Precuneus	+/− 6	− 58	46
Parieto-Occipital	+/− 51	−64	18
Supramarginal Gyrus	+/− 44	− 42	24
Precentral	+/− 44	−4	40
Middle Frontal	+/− 35	6	52
Pulvinar	+/− 20	−34	3
Fusiform Gyrus	+/− 51	− 59	−9
Superior Parietal	+/− 40	−52	− 49
Dorsal Pons	+/− 6	−36	−27
Cerebellum	+/− 46	− 53	−39
Periaqueductal Gray	−1	−26	−11

### Erenumab responder definition

Treatment response was defined as at least a 50% reduction in the frequency of migraine days during weeks 5–8 compared to the 4-week pre-treatment run-in phase as recorded in the headache diary. For months with eight or fewer days of missing diary data, data were imputed via simple proportional imputation. Larger amounts of missing diary data were imputed using diary data from the current 4-week period and the previous 4-week period combined.

### Comparing Erenumab responders to Erenumab non-responders

Participant demographics, headache characteristics, ASC-12 scores, BDI scores, MIDAS scores, headache and migraine presence at the time of research testing, and pain stimulus temperatures were compared using two-sided t-tests or Fisher exact test, as appropriate, with *p* < 0.05 being considered significant. Changes in headache frequency, migraine frequency, and ASC-12 scores were compared between erenumab responders and non-responders using two-sided t-tests with *p* < 0.05 being considered significant.

## Results

**Fig. 1:**
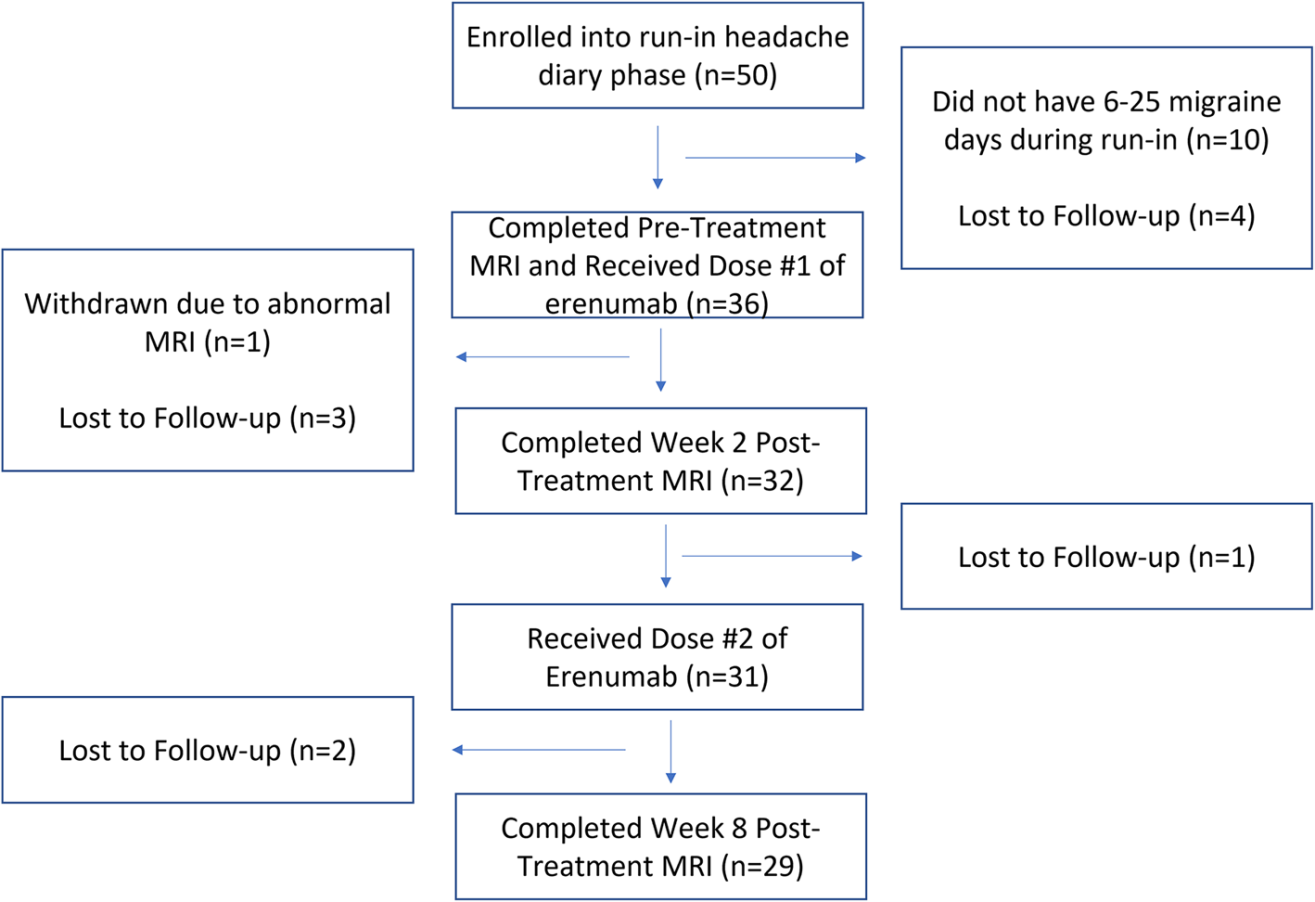
Patient Flow Through the Study. Of the 50 patients enrolled, 32 received erenumab treatment and had a pre-treatment fMRI and at least one post-treatment fMRI

The flow of participants through the study is illustrated in Fig. 1. Amongst the 50 participants who entered the run-in phase, 10 were excluded because they did not experience 6–25 migraine days during the run-in phase and 4 were lost to follow-up prior to having a baseline MRI and first erenumab treatment. Amongst the 36 remaining participants, one was withdrawn due to abnormal brain MRI findings and three were lost to follow-up. Thirty-two participants had pre- and post-erenumab fMRI and were thus included in this analysis, 29 of whom at least completed the pre-treatment and 8-week post-treatment MRI. MRI data from all three timepoints (pre-treatment, 2-week post-treatment, and 8-week post-treatment) were available from 25 participants. On average, data from 27.5 diary days was provided during the four-week run-in period and 24.6 days during the 5–8 week period.

The participants (*n* = 32) average age was 40.3 (+/− 13) and 29 were female. 31 were white, 1 was Asian, and 4 were Hispanic. Pre-treatment, they had migraine for an average of 21.9 (+/− 12.1) years, had an average of 13.8 (+/− 4.7) migraine days per month and 15.8 (+/− 4.4) headache days per month (which includes the migraine days) (Table 3). Pre-treatment, 11 had episodic migraine and 21 had chronic migraine. All participants were using migraine abortive medications. Twenty-four were using prescription abortive medications and eight were using over-the-counter abortive medications only. Nineteen of thirty-two participants had medication overuse at baseline. Migraine prescription preventive medications, in addition to erenumab, were being used by ten participants, and included propranolol (*n* = 4 participants), topiramate (*n* = 2), gabapentin (*n* = 2), venlafaxine (*n* = 2), zonisamide (*n* = 1), and amitriptyline (*n* = 1) (two patients were using two migraine preventive medications in addition to erenumab).

**Table 3:** Pre-treatment demographics, headache and migraine frequency, scores on ASC-12, MIDAS, BDI, and temperatures required to elicit moderate intensity pain. Medication overuse refers to participants who were using abortive medications more often than the thresholds defined for medication overuse by the ICHD-3 diagnostic criteria. Concurrent migraine preventive medication refers to the percentage of participants who were using migraine preventive medications in addition to erenumab. There were no differences in these pre-treatment values between participants who became erenumab responders vs. erenumab non-responders except that a larger proportion of participants who became erenumab responders were using concurrent migraine preventive medications. ASC-12 = Allodynia Symptom Checklist 12; MIDAS = Migraine Disability Assessment; BDI = Beck Depression Inventory

	All Participants (*n* = 32)	Erenumab Responders (*n* = 18)	Erenumab Non-Responders (*n* = 14)	*p*-value (responders vs. non-responders)
Age, mean (SD)	40.3 (13)	41.9 (13)	38.3 (13)	0.45
Female, percentage	91%	89%	93%	0.99
Headache Day Frequency/ 28 days, mean (SD)	15.8 (4.4)	15.8 (4.8)	15.7 (3.9)	0.96
Migraine Day Frequency/ 28 days, mean (SD)	13.8 (4.7)	14.8 (5.1)	12.6 (4.2)	0.20
Years with Migraine, mean (SD)	21.9 (12.4)	22.6 (12.9)	20.8 (12.2)	0.70
Medication Overuse, percentage	59.4%	66.7%	50%	0.47
Concurrent Migraine Preventive Medication, percentage	31.3%	55.6%	0%	.001
ASC-12 scores, mean (SD)	4.7 (3.1)	4.5 (3.1)	4.9 (3.2)	0.70
Thermode Temperature Causing Moderate Pain, mean (SD), ^0^C	45.8 (2.4)	46.0 (2.8)	45.6 (1.8)	0.63
MIDAS scores, mean (SD)	35.9 (27)	38 (31)	33 (21)	0.61
BDI scores, mean (SD)	5.8 (4.8)	5.6 (4.3)	6.1 (5.6)	0.80

Amongst all pre-treatment research visits for testing, headache was present during 15/32 (46.9%) visits and migraine was present on 5/32 (15.6%) visits. Amongst participants who went on to be erenumab-responders, headache was present during 9/18 (50%) pre-treatment visits compared to those who did not go on to be erenumab responders in whom headache was present during 6/14 (42.9%) pre-treatment visits (*p* = 0.73). Amongst participants who went on to be erenumab-responders, migraine was present during 4/18 (22.2%) pre-treatment visits and during 1/14 (7.1%) pre-treatment visits for those who went on to be erenumab non-responders (*p* = 0.35) .

Amongst all post-treatment research visits for testing, headache was present during 12/59 visits and migraine was present during none of the visits. Amongst erenumab responders, headache was present during 5/59 (8.5%) of post-treatment visits compared to 7/59 (11.9%) post-treatment visits among erenumab non-responders (*p* = 0.76).

Eighteen (56%) participants were erenumab responders and 14 (44%) were non-responders. Changes in headache day frequency, migraine day frequency, and ASC-12 scores are shown in Table 4. There were no differences in post-treatment thermode temperatures required to cause moderate intensity pain. Post-treatment amongst all participants the average temperature was 45.3 °C (+/− 2.2 °C); 45.6 °C (+/− 2.3 °C) in erenumab responders vs. 45.0 °C (+/− 2.2 °C) in erenumab non-responders (*p* = 0.32).

**Table 4:** Changes in headache frequency, migraine frequency, and ASC-12 scores. Change in headache and migraine frequency compares weeks 5–8 after the first erenumab treatment to the 4-week pre-treatment period. Change in ASC-12 scores compares those obtained during the research visit at 8 weeks after the first erenumab treatment to those collected just before the first treatment. ASC-12 = Allodynia Symptom Checklist 12

	All Participants (*n* = 32)	Responders (*n* = 18)	Non-Responders (*n* = 14)	p-value (responders vs. non-responders)
Change in Headache Day Frequency/ 28 days, mean	− 7.1	−10.1	− 3.4	< 0.001
Change in Migraine Day Frequency/ 28 days, mean	− 6.9	− 10.3	−2.4	< 0.001
Change in ASC-12 scores, mean	− 1.6	−3.4	0.8	< 0.001

### Adverse events

AEs were reported by 25 participants who reported a total of 36 AEs. There were no serious AEs. Eighteen were considered mild, 13 moderate, and 5 severe. Fourteen AEs were considered unrelated to study medication (dizziness × 2, and one each of flank pain, loss of balance, weight gain, twitching of arms, ear infection, urinary tract infection, agitation, communication issues, insomnia, COVID-19 infection, allergic reaction, West Nile Virus infection), 5 were unlikely to be related (itching × 2, and one each of dizziness, skin sensitivity, tinnitus), 1 was possibly related (worsening headache), 15 were probably related (constipation × 14, abdominal pain × 1), and 1 was definitely related (injection-site erythema). Two participants withdrew from the study due to adverse events, including one patient who had constipation and abdominal pain and one patient who reported worsening headaches.

### Pain-induced brain activations

**Fig. 2:**
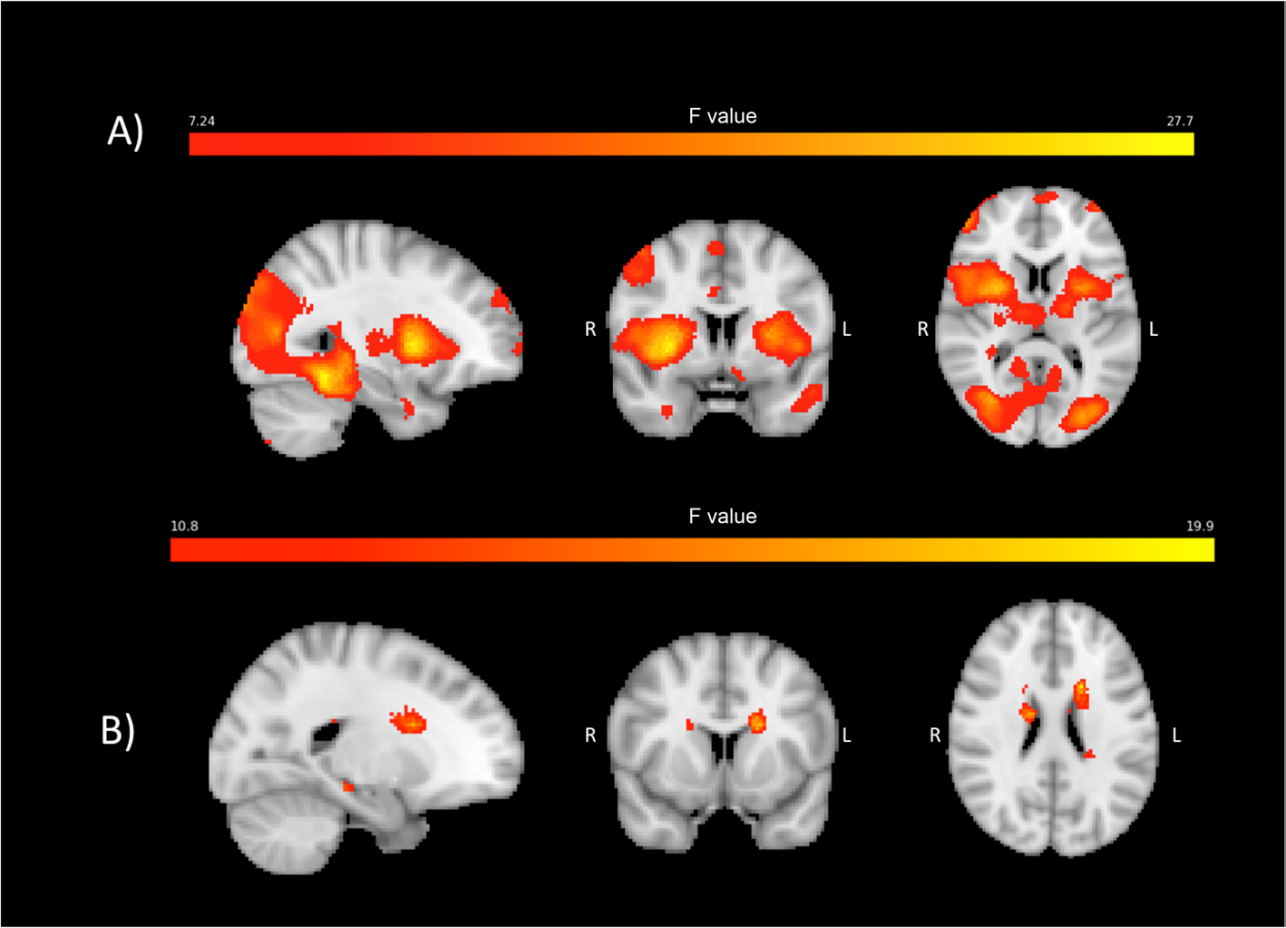
Main effect of painful heat applied to the left forearm at baseline (i.e. pre-treatment). A) immediate BOLD response to painful stimulation (measured starting with the MRI frame during which the painful heat stimulus began and ending 12.5 s later). B) delayed BOLD response to painful stimulation (measured starting 12.5 s after onset of painful heat stimulus and ending 12.5 s later). Hotter colors represent stronger activation

The main effect of painful heat stimulation to the left forearm at the baseline visit is demonstrated in Fig. 2. This main effect demonstrates activation of ‘pain matrix’ regions, i.e., those brain regions that have consistently shown to be activated by painful stimulation and provides evidence that the pain stimuli resulted in the expected brain activations.

**Fig. 3:**
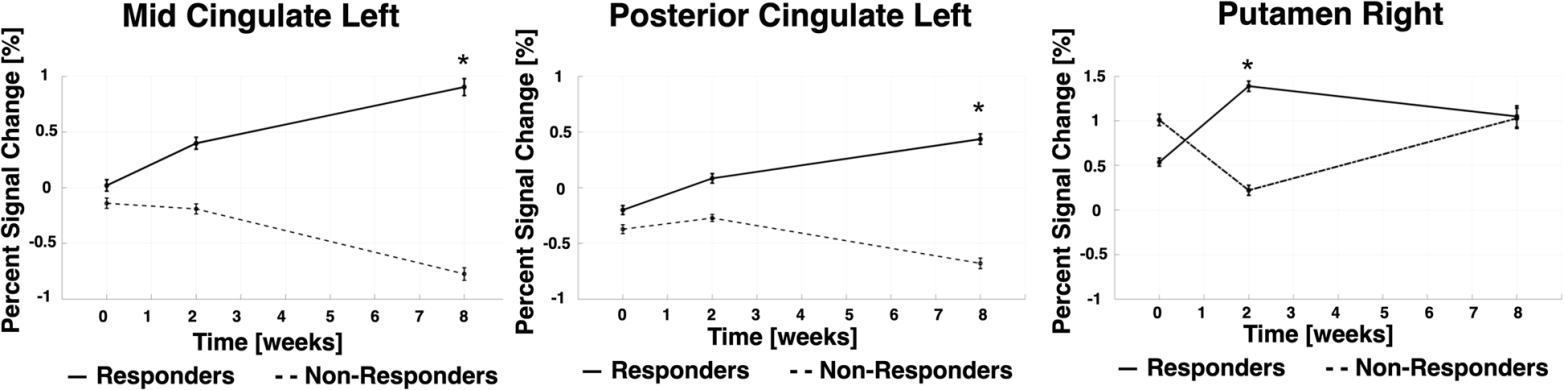
Pain-induced BOLD activations measured immediately after onset of painful stimuli, comparing erenumab-responders to non-responders. The “immediate” BOLD activations were measured starting with the MRI frame during which the painful heat stimulus began and ending 12.5 s later. The group-visit effect with *p* < 0.005 with voxel forming threshold above 50 is shown for 3 regions. The longitudinal contrast averages in these regions are shown with error bars representing standard error. Mid = middle

When comparing erenumab responders to non-responders, at post-treatment visits there were significant differences in brain activations occurring immediately after onset of painful stimuli in the left middle cingulate, left posterior cingulate, and right putamen (Fig. 3).

The left middle cingulate and left posterior cingulate showed significant differences in pain-induced activations between erenumab-responders and non-responders at visit 3 (*p* < 0.001) whereas the putamen showed a transient difference at visit 2 (*p* = 0.002). In all three regions the baseline activation is similar between groups.

**Fig. 4:**
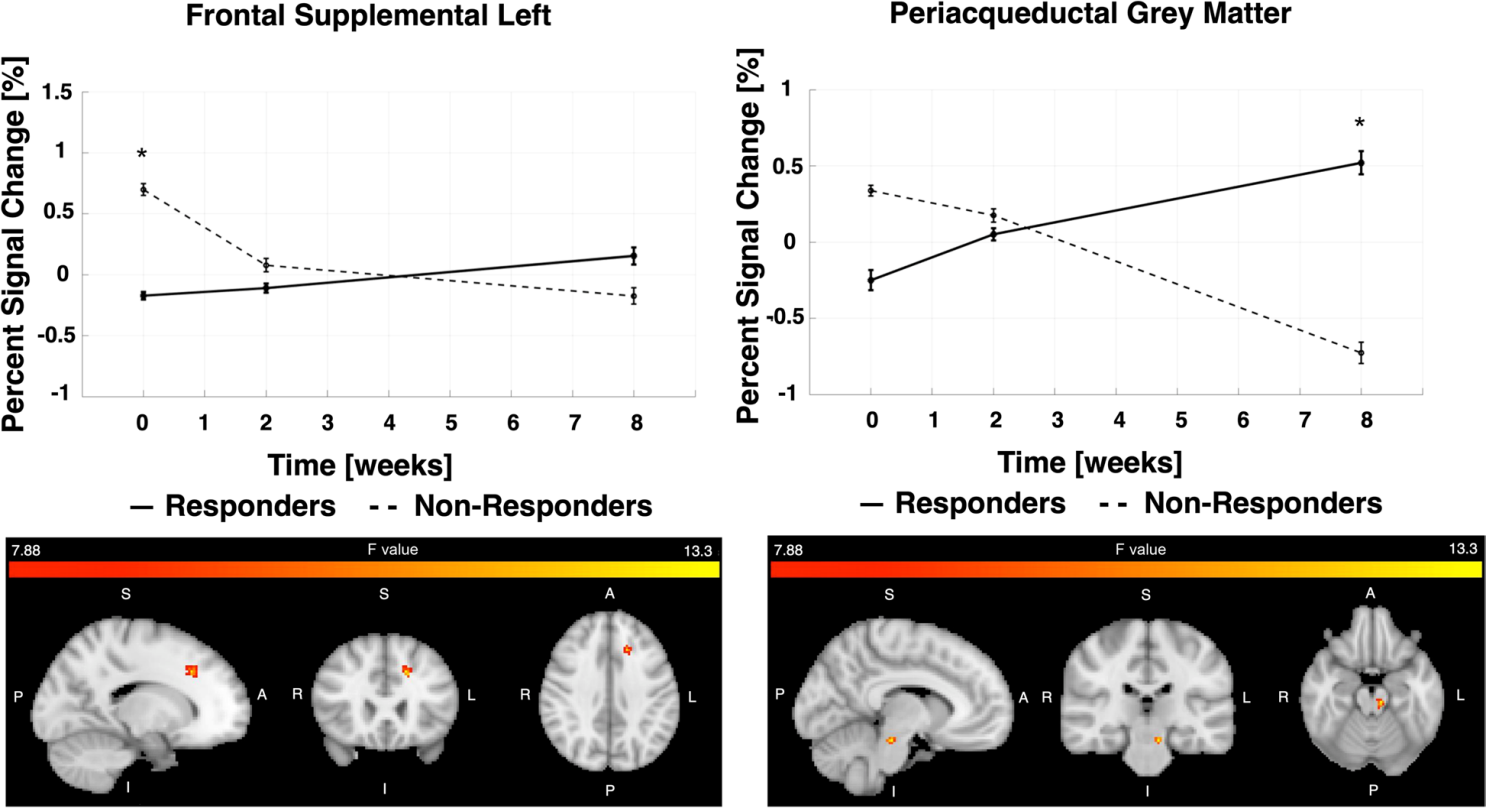
Delayed pain-induced BOLD activations in response to painful stimuli, comparing erenumab-responders to non-responders. The “delayed” response to pain was measured starting 12.5 s after onset of painful heat stimulus and ending 12.5 s later. Areas shown had significance of *p* < 0.005 and cluster forming threshold of 25 voxels. Longitudinal average contrasts are plotted per visit with standard error bars

Analyses of delayed BOLD activations in response to painful stimulation demonstrated differences between erenumab-responders vs. non-responders for the periaqueductal gray and left frontal supplemental area (Fig. 4). The periaqueductal gray matter response to pain was greater in erenumab-responders vs. non-responders at the 3rd visit (*p* = 0.003). The frontal supplemental region had a greater response in the 1st visit in non-responders compared to responders (*p* = 0.005).

### Resting state functional connectivity

**Fig. 5:**
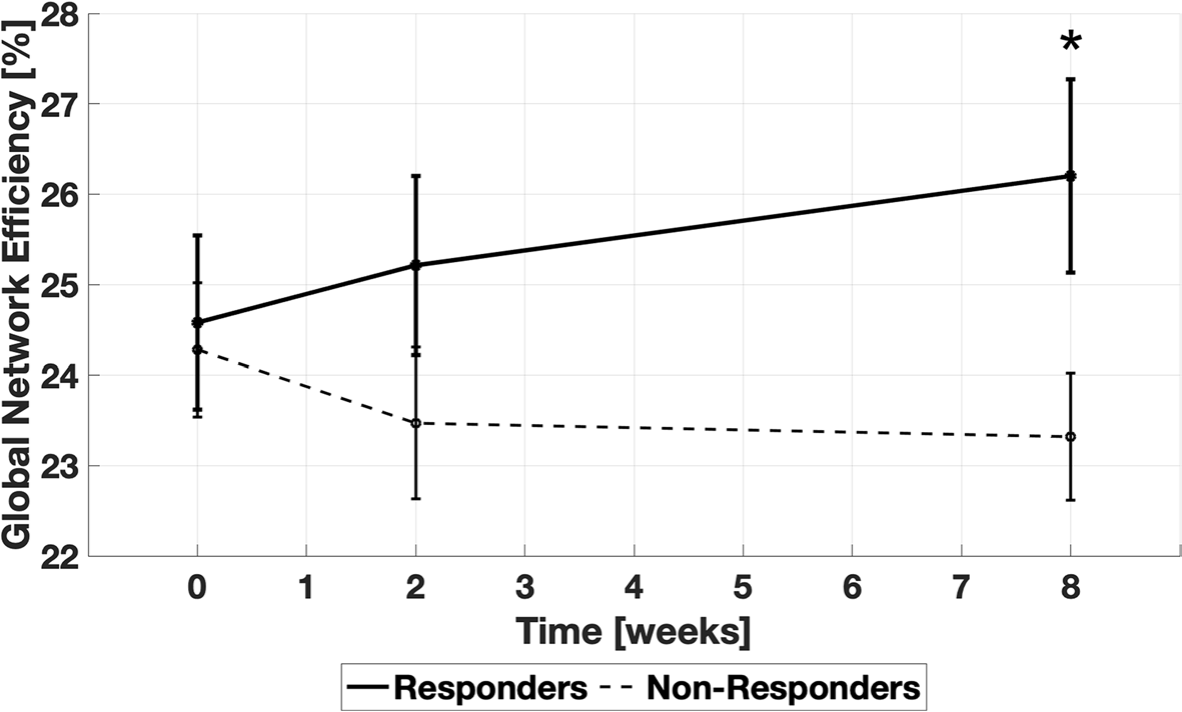
Longitudinal changes of global efficiency amongst 63 ROIs that participate in migraine and/or pain processing for erenumab responders and non-responders. At 8 weeks after the first erenumab treatment the global efficiency was higher in the erenumab-responders compared to the non-responders. Error bars represent standard error

Pre-treatment, the global efficiency amongst all a priori selected ROIs did not differ between erenumab responders (24.6% ± 1%) vs. non-responders (24.3% ± 0.7%, *p* = 0.81). However, there was a difference in global efficiency at 8 weeks after the first erenumab treatment in responders (26.2% ± 1.1%) vs. non-responders (23.3% ± 0.7%, *p* = 0.04). The longitudinal measures of global efficiency are shown in Fig. 5.

The local network measures for each ROI that differed between erenumab-responders vs. non-responders are shown in Table 5. Differences for several network measures were identified, including regional efficiency, betweenness, clustering coefficient, node degree, and modularity.

**Table 5:**
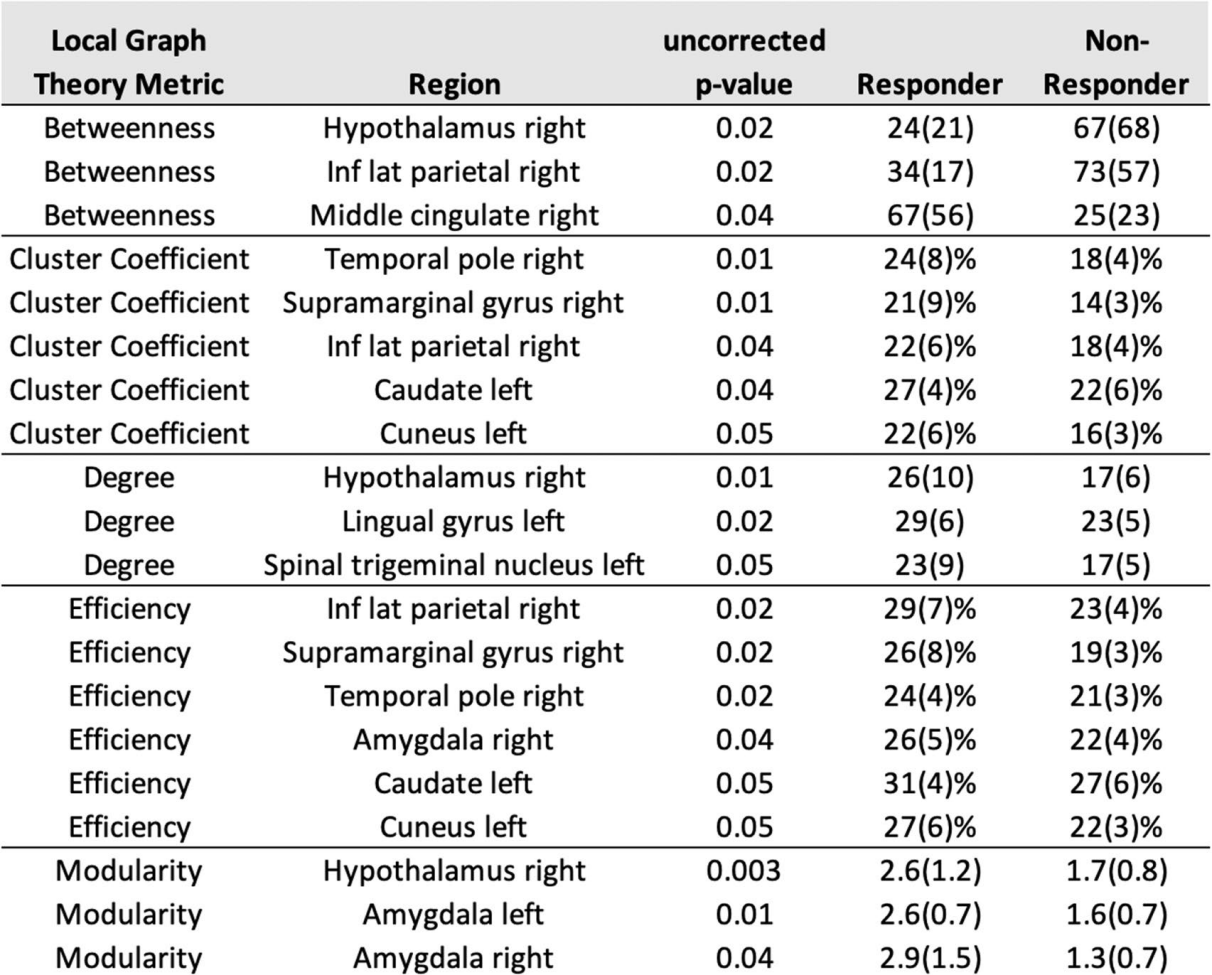
Regional network differences between erenumab responders and non-responders at 8 weeks after the first erenumab treatment. Shown for all uncorrected *p*-values of 0.05 or less. Numbers in parentheses are standard deviations. Inf = inferior; Lat = lateral

The pairwise differences in ROI-to-ROI correlations between erenumab-responders vs. non-responders at 8 weeks following the first erenumab treatment are shown in Table 6. These functional connections included regions such as the supramarginal gyrus, inferior lateral parietal, hypothalamus, temporal pole, middle temporal, middle occipital, and middle frontal lobes, dorsolateral and ventromedial prefrontal cortices, and the pulvinar.

**Table 6:**
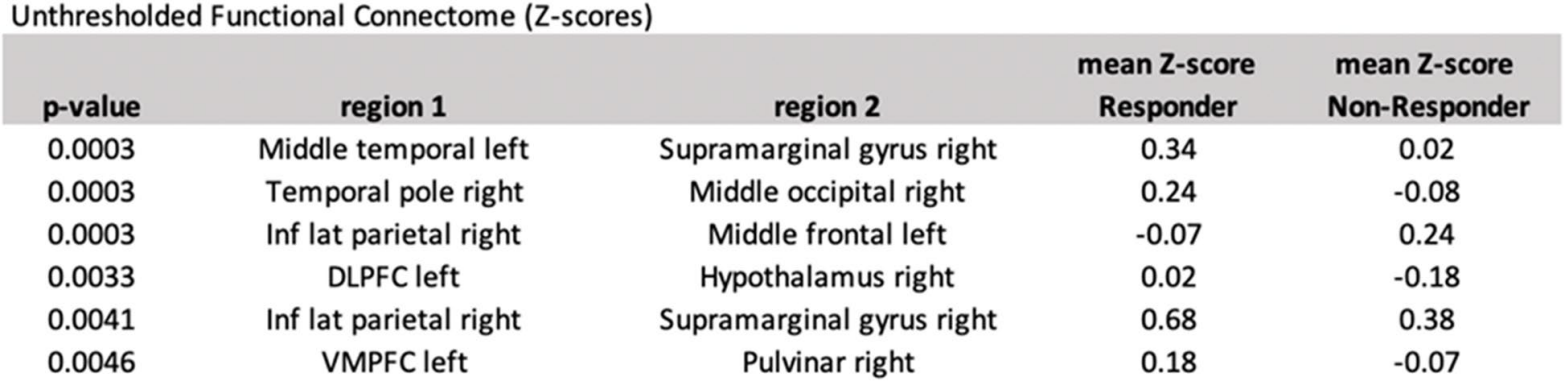
ROI-to-ROI functional connectivity differences between erenumab responders and non-responders at 8 weeks after the first erenumab treatment. Results with uncorrected *p*-values of 0.005 or less are shown. Inf = inferior; lat = lateral; DLPFC = dorsolateral prefrontal cortex; VMPFC = ventromedial prefrontal cortex

## Discussion

The main finding of this study is that effective migraine treatment with erenumab is associated with changes in brain activations in response to extracranial thermal pain and with changes in resting-state functional connectivity. fMRI differences between erenumab-responders vs non-responders were seen within 8 weeks of initiating erenumab treatment, with some differences starting to emerge at 2 weeks. Brain regions impacted across the different analyses in this study included the hypothalamus, inferior lateral parietal, temporal pole, supramarginal gyrus, amygdala, and periaqueductal gray, amongst others. As discussed further below, these regions are known to have important roles in migraine pain processing including their likely roles in migraine attack generation, pain perception, pain modulation, and multisensory integration.

### Pain-induced activations

Our pain-induced activation analyses identified differences between erenumab responders vs. non-responders in activations of regions in the cingulate cortex, putamen, frontal lobe, and periaqueductal gray matter. Brain activations occurring immediately (i.e., within 12.5 seconds) after painful stimuli and delayed activations occurring between 12.5 s to 25 seconds after painful stimuli were investigated. Prior studies have demonstrated a biphasic hemodynamic brain response to painful heat stimuli, consisting of a stimulus-locked response and a second peak delayed by about 12.5 seconds [25]. It has been suggested that the initial BOLD response might be related to pain processing via myelinated A-delta fibers, while the delayed BOLD response could be associated with processing via the slower C fibers [25, 41]. It has also been theorized that the initial BOLD response might represent a fast, non-conscious processing of pain that helps to quickly determine the threat level, while the delayed response might represent a more conscious processing of the painful stimulus [25, 41, 42]. Our study identified differences in immediate and delayed BOLD responses to pain when comparing erenumab-responders vs. non-responders.

For immediate BOLD responses, there were differences in the middle and posterior cingulate at 8 weeks following the first erenumab treatment and in the putamen only at 2 weeks following the first erenumab treatment. Changes in BOLD responses in the middle cingulate and posterior cingulate were already seen at 2 weeks after the first erenumab treatment, but the differences between erenumab-responders and non-responders were not significant until the eight-week MRI. The middle cingulate participates in rapid behavioral adaptive responses to the threat associated with pain, as well as cognitive and affective components of pain processing [43, 44]. A prior fMRI study of migraine demonstrated a strong positive correlation between pain-induced activation in the middle cingulate and headache frequency (r = 0.627, *p* = .001), providing relatively strong evidence for its role in migraine [24]. In our study, it is possible that the increase in pain-induced activation of the middle cingulate amongst erenumab-responders is due to the pain stimulus as being perceived as more novel and thus as a greater threat compared to the erenumab non-responders who are experiencing more frequent pain, with the painful stimulus thus losing its novelty. The posterior cingulate, a key region of the default mode network, participates in self-referential processing of stimuli, including externally generated pain [45]. Numerous pain and migraine studies have demonstrated atypical functional connectivity of default mode network regions [46–49]. A prior migraine study demonstrated that migraine improvement (i.e. reduced time with headache) was associated with cortical thickness changes in the left posterior cingulate [50]. The putamen participates in sensory-discriminative aspects of pain processing including the determination of pain sensitivity [51]. Prior migraine studies have demonstrated atypical functional connectivity and structure of the putamen [52, 53]. In our study, differences in putamen activation were detected at two-weeks following the first erenumab treatment, but not at 8 weeks, a pattern that is difficult to interpret.

Delayed BOLD response differences between erenumab-responders and non-responders were identified in the periaqueductal gray at 8 weeks following initiation of erenumab treatment and in the frontal supplementary area prior to starting erenumab (and thus not related to treatment). The periaqueductal gray is a key region of the pain modulating pathway, predominantly involved in pain inhibition. Numerous studies have identified atypical periaqueductal gray structure and function associated with migraine, the extent to which might correlate with migraine disease severity [54–58]. Less effective pain modulation by the periaqueductal gray could be a mechanism by which those with migraine experience more severe pain, allodynia, and increased frequency of migraine attacks [54–57]. Increased pain-induced activation of the periaqueductal gray amongst erenumab-responders might reflect a stronger pain-inhibitory response.

Overall, our pain activation studies suggest that effective erenumab treatment is associated with changes in cognitive, affective, sensory-discriminative, and modulating aspects of pain processing.

### Resting-state functional connectivity

Our resting-state functional connectivity analyses included investigation of graph theory network parameter differences and ROI-to-ROI static functional connectivity differences between erenumab-responders vs. non-responders. Graph theory provides a method for quantitatively describing the topological organization of brain networks [38]. Graph theory measures in our analyses included global and regional efficiency, betweenness, clustering coefficient, node degree, and modularity [39, 40]. Efficiency is the inverse of the minimum path length between an ROI and all other ROIs in a network [38]. Global and local efficiency reflect a network’s ability to transmit information at the global and local levels [40]. Betweenness of a ROI is the number of shortest paths between any two ROIs that run through the ROI [38]. Clustering coefficient describes the local connectedness of a network and is calculated by determining the number of connections that a ROI has with its immediate neighbors divided by all of its possible connections [38]. Node degree is a measure of the number of connections to an ROI [40]. Modularity measures how connected ROIs are to members of their own group, identifying subnetworks within a larger network.

Erenumab responders had an increase in global efficiency from pre-treatment to eight-weeks post-treatment and greater global efficiency at eight-weeks compared to erenumab non-responders. An increase in global efficiency suggests that erenumab-response was associated with an improvement in the ability of the studied regions, those that participate in various aspects of pain processing and modulation, to functionally communicate on a global scale. At eight-weeks post-treatment, compared to erenumab non-responders, responders had ROIs with higher efficiency, cluster coefficient, node degree, and modularity, findings suggesting a greater ‘small world’ quality of the network. Regions most strongly highlighted by these differences included those in the hypothalamus, amygdala, and inferior parietal lobe. The hypothalamus likely plays an important role in the generation of migraine attacks [59, 60]. The amygdala contributes to affective and attentional responses to pain and pain modulation [61]. The inferior parietal region, like the posterior cingulate discussed above, is a key region of the default mode network [62]. Other regions included the temporal pole, supramarginal gyrus, caudate, cuneus, trigeminal nucleus, lingual gyrus, and middle cingulate.

ROI-to-ROI differences in functional connectivity at 8 weeks between erenumab-responders vs. non-responders included regions that were also identified as having differences in graph theory network measures, including the supramarginal gyrus, inferior lateral parietal, hypothalamus, and temporal pole. Additionally, several other regions were involved in these functional connections including regions in the middle temporal, middle occipital, and middle frontal lobes, dorsolateral and ventromedial prefrontal cortices, and the pulvinar.

### Study results in context of prior studies

Prior studies have interrogated the impact of migraine treatment on brain functional connectivity and brain activations. Krebs and colleagues demonstrated that treatment with sphenopalatine ganglion blocks was associated with changes in functional connectivity amongst salience network and executive network regions [8]. Russo and colleagues investigated pain-induced brain activations before and after 60 days of treatment with external trigeminal neurostimulation [9]. Neurostimulation treatment was associated with a reduced BOLD response in the anterior cingulate cortex. Acupuncture treatment has been associated with an increase in periaqueductal gray functional connectivity with anterior cingulate cortex among individuals who have migraine without aura [63]. A pre-treatment and 2–3 week post-treatment study of galcanezumab, a CGRP ligand mAB, for migraine demonstrated that galcanezumab decreased hypothalamic activation in response to nociceptive trigeminal stimulation to a greater extent in galcanezumab-responders vs. non-responders [12]. There were also responder-specific decreases in BOLD activation in the inferior parietal lobule, insula and parahippocampal gyrus. Spinal trigeminal nucleus functional connectivity changes from the pre-treatment to post-treatment scans were interrogated for all treated patients and demonstrated weakened connectivity with hypothalamus and superior temporal gyrus and stronger connectivity with the cerebellum, middle temporal gyrus, and insula at the post-treatment timepoint. The study most closely related to ours investigated the impact of erenumab on brain activations in response to nociceptive trigeminal stimulation [10]. In that study, 27 individuals with migraine underwent fMRI prior to and 2 weeks after treatment with 70 mg of erenumab. During the fMRI paradigm, intranasal ammonia was used as a painful stimulus. 63% of participants were considered erenumab treatment responders, which was defined as at least a 30% reduction in headache days during the first month following treatment. Amongst all patients there were post-treatment decreases in pain-induced activations in the thalamus, lingual gyrus, middle temporal gyrus, operculum, and cerebellum. Compared to non-responders, erenumab-responders had a significant reduction of activation in the hypothalamus, insula, and cerebellum. Analysis of hypothalamic functional connectivity amongst all treated patients demonstrated a reduction in connectivity strength with the temporal lobe, hippocampus, parahippocampus, fusiform gyrus, cerebellum, red nucleus, and spinal trigeminal nucleus, and an increase in connectivity strength with the anterior insula. This study and ours complement one another, both demonstrating changes in pain-induced activation and resting functional connectivity associated with erenumab treatment and response. Our study adds to the literature since it determined treatment response during weeks 5–8 after starting treatment, studied 140 mg of erenumab rather than 70 mg, investigated early (i.e. 2 weeks) and later (i.e. 8 weeks) fMRI changes after initiating treatment, utilized two treatments with erenumab rather than one, used an extra-trigeminal painful heat stimulus for the event-related paradigm, and interrogated graph theory network measures of functional connectivity.

### Relationship between fMRI changes and Erenumab treatment

How treatment with erenumab is associated with changes in pain-induced brain activations and functional connectivity is a matter of debate. It is perhaps unlikely that the small amount of erenumab that might cross the blood-brain barrier could exert a meaningful central effect and have a direct impact on brain processing of painful stimuli. Alternatively, erenumab might alter brain pain processing indirectly, via its impact on peripheral structures such as the trigeminal ganglia, trigeminal nerves, or dura mater [16–18]. Finally, it is possible that the changes in pain processing demonstrated in this study are attributable to the reduction in migraine days associated with erenumab response, but not specifically attributable to the mechanisms by which erenumab exerts therapeutic effects.

### Study considerations and limitations

Considerations and limitations of our study include: 1) Although the number of MRIs completed and included in this study is relatively large (*n* = 86), the number of erenumab-responders (*n* = 18) and non-responders (*n* = 14) is relatively small. Larger sample sizes might allow for more stringent statistical corrections for multiplicity. 2) Our study is not able to determine if the pre- to post-erenumab changes in pain-induced brain activations and functional connectivity are directly attributable to erenumab or if there would be similar findings associated with reductions in migraine frequency regardless of the specific reason for such a reduction. Optimally, future studies would include migraine treatments that work via different mechanisms and individuals who have longitudinal reductions in migraine frequency in the absence of treatment. 3) Inclusion of a healthy control group would allow for better interpretation of the pre-to-post treatment changes, whether the changes are consistent with a “normalization” of brain function or adaptive changes, for example. 4) Neuroimaging research studies use different statistical corrections and cluster forming thresholds for determining the significance of results. More stringent methods increase the likelihood for type II error, while less stringent methods increase the likelihood for type I error. The approaches taken in this study should be considered when interpreting the results. Like with all neuroimaging studies, replication of results would further strengthen the assessment of their validity. 5) The sample size of research participants included in this study prevented us from performing additional subgroup analyses, such as differences that might be present based on headache frequency (e.g. episodic migraine vs. chronic migraine), participant sex, use of concurrent migraine preventive medications, and frequency of using migraine as-needed medications. 6) Prior to starting erenumab, a larger proportion of participants who became erenumab responders were concurrently taking other migraine preventive medications. The use of concurrent migraine preventive medications could have an impact on pre-treatment fMRI comparisons between responder and non-responder groups but is unlikely to have impacted differences that were first seen at 2 weeks or 8 weeks following initiation of erenumab. 7) We did not limit the use of medications within 48 hours of the MRI and QST. Thus, some participants had used abortive medications within that time-period. No participants were using opiates, which might directly impact pain sensation during QST and thermal stimulation. 8) QST and fMRI results could be impacted by the participants headache and migraine state, meaning that findings might differ according to whether headache or migraine is present during the test. However, in our study and as presented in the Results, the frequency of testing during headache or migraine was similar between erenumab-responders vs. non-responders and there were few post-treatment QSTs and MRIs collected during headache and none during migraine. Thus, there was not ample justification or sample sizes for analyzing QST and MRI data according to headache and migraine status. 9) Future fMRI studies could use other timepoints for determining treatment response, such as the 9–12-week period after starting erenumab. It is possible that longer durations of response to erenumab could be associated with more substantial changes in fMRI measurements.

## Conclusions

Compared to erenumab non-responders, response to erenumab for migraine prevention is associated with post-treatment differences in pain-induced brain activations and resting state functional connectivity. Whether direct or indirect, results suggest that erenumab has effects on brain function, likely impacting central nervous system migraine mechanisms.

## Data Availability

The datasets used and/or analyzed during the current study are available from the corresponding author on reasonable request.

## Abbreviations

ASC-12: Allodynia Symptom Checklist 12
BDI: Beck Depression Inventory
BOLD: Blood-oxygen-level-dependent
CGRP: calcitonin gene-related peptide
DLPFC: dorsolateral prefrontal cortex
fMRI: functional magnetic resonance imaging
FWHM: full width half maximum
GLM: general linear model
Inf: inferior
Lat: lateral
mAb: monoclonal antibody
Mid: middle
MIDAS: Migraine Disability Assessment
MNI: Montreal Neurological Institute
QST: quantitative sensory testing
ROI: region of interest
SWE: sandwich estimator
VMPFC: ventromedial prefrontal cortex
